# Attitudes of Chinese health sciences postgraduate students’ to the use of information and communication technology in global health research

**DOI:** 10.1186/s12909-019-1785-6

**Published:** 2019-10-09

**Authors:** Kaiyong Huang, Abu S. Abdullah, Zhenyu Ma, Dilshat S. Urmi, Huimin He, Lisa Quintiliani, Robert H. Friedman, Jun Yang, Li Yang

**Affiliations:** 1 School of Public Health, Guangxi Medical University, Nanning, 530021 Guangxi Province China; 2 Boston Medical Center, Boston University School of Medicine, Boston, MA 02118 USA; 3 Duke Global Health Institute, Duke University, Durham, North Carolina USA; 4 Global Health Program, Duke Kunshan University, Kunshan, 215347 Jiangsu Province China; 5 School of Information Management, Guangxi Medical University, Nanning, 530021 Guangxi Province China

**Keywords:** Information and communication technology, ICT, Health sciences students, Global health research, Training curriculum

## Abstract

**Background:**

Information and communications technology (ICT) has been suggested as an important tool for improving global health education and building research capacity in developing countries. However, the existing curricula do not have adequate emphasis on global health research and training. This study was carried out to examine health sciences postgraduates’ attitudes and practices regarding curriculum for ICT use in global health research and training in China.

**Methods:**

A cross-sectional study was conducted among health sciences postgraduates from six universities in southern China, during December 2016 to March 2017. A self-administered online questionnaire was used to collect data through an online survey platform. Data were analyzed using SPSS for Windows 13.0.

**Results:**

A total of 1065 participants successfully completed the questionnaires. More than 90% of the students have not had any training about ICT, three quarters have not taken an online course, and 31% of the students do not use ICT in their current research. More than 65% thought that, in an ICT research training curriculum, it was important to learn: ICT utilization related knowledge, ICT research methods/resources, knowledge of databases, ways of data use and acquisition, and informatics search methods (ICT users compared to non-users were more likely to agree to these learning components (all *p* <  0.05)). Many of the respondents used or planned to use mobile phones (80%), Internet (59%), use computer and WeChat (> 40%), and QQ (a popular chat tool in China) (30%) as ICT tools in research activities. ICT users compared to non-users were more likely to consider using ICT and/or biomedical informatics methods in decision-support or support for information seeking, healthcare delivering, academic research, data gathering, and facilitating collaboration (all *p* <  0.05).

**Conclusions:**

The findings of this study showed that ICT utilization was very important to health sciences postgraduates for their research activities in China, but they lacked ICT-related training. The results suggested the need for specialized curriculum related to ICT use in global health research for health sciences postgraduates in China.

## Background

Information and Communication Technology (ICT) can be defined as a collection of technologies and applications that are used to process, store and disseminate information to a wide variety of users or clients [1]. ICT has greatly impacted peoples’ daily lives, through providing faster and more convenient communication, easier acquisition of information and its ability to provide better quality of life [2]. In developed countries, ICT has been widely used in various industries, such as petrochemical industry, agriculture, education, environmental monitoring, public health services, especially in health care and health-related research, making great advances. For instance, an ICT-based centralized monitoring system was used to monitor immunosuppressive medication use in kidney transplant recipients. It will alert both medical and staff patients with texts and pill box alarms if there is a dosage/dosing time error or a missed dose [3]. Being a powerful tool, ICT also offers developing countries with emerging opportunities to achieve their development goals [4]. In most developed countries, ICT tools used in health education and research have been embedded in national strategies, where governments set guidelines that promote the use of ICT [1]. However, in most developing countries, the use of ICT is quite limited.

In recent years, the application of ICT tools is increasingly becoming an absolutely vital component of the learning and teaching environment in many medical colleges [5]. With the benefits of ICT in global health such as online medical consultation, diagnosis and treatment [6], opportunity to exchange health information [7], access to health training [8], access to social network analysis and mining [9] and support to health policies decision-making [10], ICT has been suggested as an important tool for improving global health education, increasing collaboration in global health research, and building research capacity in low- and middle-income countries [1, 11]. Due to shortages of funding, lack of proper policies, lack of professionals and limited Internet access, the use of ICT in health education and training is insufficient in developing countries [4]. Therefore, in developing countries, faculty members working on health education and health training activities are often required to make major changes in the technology used in their teaching and training without sufficient support or knowledge of what curriculum components are most desired among students [1]. Although more and more curricula for ICT use are carried out in educational and research fields in some developing countries, such as China, Russia, Brazil and India, the curricula for ICT use in health research fields remains considerably insufficient compared to developed countries [4, 12, 13].

In addition, there are few studies regarding the use and demand of ICT training in health education and global health research in China. The purpose of this study was to examine Chinese health sciences postgraduates’ attitudes and practices regarding curriculum for ICT use in global health research and training in China.

## Methods

### Study design, sample and setting

This study used a descriptive, cross-sectional design. Participants were postgraduate students of health sciences programs from the six major medical universities in southern China covering three provinces, Guangxi, Guangzhou and Yunnan, and one municipality directly under the central government (Shanghai) [11]. These universities are located in three cities in less developed regions (Kunming, Nanning and Guilin) and two cities in developed regions (Shanghai and Guangzhou). The six universities were Guangxi Medical University (GXMU, Nanning), Guangxi University of Chinese Medicine (GXUCM, Nanning), Guilin Medical University (GLMU, Guilin), Kunming Medical University (KMMU, Kunming), Guangzhou Medical University (GZMU, Guangzhou), and Fudan University (FDU, Shanghai) [1].

In general, in these universities, the use of technology in education and training is not widely implemented. Lectures are still mostly delivered using power point slides with assignments (if any) collected in hard copies. Campus wide internet facilities is available within the designated areas or buildings. Computer labs are available but there are limitations to meet the needs for all students.

### Data collection

A self-administered online questionnaire was used to collect data through Wen Juan Xing (a professional online survey platform, see Additional file 1), during December 2016 to March 2017. The questionnaire was developed based on our earlier qualitative study among the health sciences graduate students [11], and revised based on a pilot study conducted among 10 participants. These 10 participants were not included in the final study. The questionnaire collected data on: demographic characteristics (gender, age, university attended, research field or interested field, whether or not taking an online course, and having training about ICT), questions on ICT use, use of ICT tools, attitudes towards ICT use, and practices regarding ICT curriculum [11]. ICT use was defined as using any ICT and/or biomedical informatics tools in their current research activities. Information about the study was provided at the beginning of the questionnaire, and consent to participate was obtained on the first page of the web-based questionnaire through a check box agreement. To compensate for their time, each participant obtained a cash incentive of RMB 5 (0.7 USD). This study was approved by the Ethics Committee of the Guangxi Medical University (No. IRB-SPH-2015: 009). The data collected were used exclusively for the purpose of the study.

### Analysis

We began by exporting all the questionnaire data from survey platform. Quantitative variables were described as means and standard deviations if they were normally distributed. Frequencies and percentages were used to describe the qualitative variables. To examine differences between the participants who were using ICT and those who were not using ICT, a Chi-square test was performed. A *p*-value of < 0.05 (two-tailed) was considered statistically significant. We analyzed data with SPSS for Windows 13.0 (IBM, New York, NY, USA).

## Results

### Characteristics of study sample

A total of 1065 participants successfully completed the questionnaires. Incomplete questionnaires were not included. Of the respondents, 54% were female and 58% were between ages of 22 and 25. Overall, 69% came from Guangxi Medical University, and the most frequently identified research field was clinical medicine (43%). Participants’ current disciplines ranged from clinical medicine (49%), public health (17%), pharmacy (11%), medical information and management (11%), and nursing (11%). More than 90% of the students did not have any training in any aspect of ICT or biomedical informatics, and three quarters of students have not taken an online course (Table 1).

**Table 1:** Demographic characteristics of the study population (*N* = 1065)

Variables	*N*	%
Gender		
Male	489	46%
Female	576	54%
Age		
22–25	622	58%
26–30	304	28%
Above 30	139	14%
Universities		
Guangxi Medical University (GXMU, Nanning)	733	69%
Guangxi University of Chinese Medicine (GXUCM, Nanning)	91	8%
Guilin Medical University (GLMU, Guilin)	90	8%
Guangzhou Medical University (GZMU, Guangzhou)	49	5%
Kunming Medical University (KMMU, Kunming)	51	5%
Fudan University (FDU, Shanghai)	51	5%
Current Disciplines		
Clinical Medicine	527	49%
Public Health	180	17%
Pharmacy	115	11%
Nursing	127	12%
Medical Information and Management	116	11%
Research fields, current or interested fields		
Biomedicine	171	16%
Clinical Medicine	455	43%
Health Communication	74	7%
Biomedical, Medical or Public Health Informatics	86	8%
Population health and/or public health research	81	8%
Epidemiology/clinical epidemiology	79	7%
Health services research	62	6%
Behavioral/social science research	44	4%
Others	13	1%
Have any of you had training, either formal or informal, in any aspect of ICT or biomedical informatics?		
Yes	72	7%
No	993	93%
Have you ever taken an online course?		
Yes	269	25%
No	796	75%

### Attitudes towards ICT

Overall, 740 students (69%) used ICT and 325 students (31%) did not use ICT in their current research activities. Of the 1065 respondents, more than 65% expressed that it was very important or essential in an ICT research training curriculum include the following components: “Learning what researchers need to know about ICT to be efficient and effective researchers”, “Most useful ICT research methods/resources across the research process and where/how to access them”, “Fundamentals of how databases are structured, attributes of data, ways data can be stored/retrieved and how pieces of data relate to each other”, “Using data from electronic health records in research”, “Using informatics search methods to help develop research protocols”, and “Performing personal computer hygiene” (ICT users compared to non-users were more likely to agree to these issues (*p* <  0.05)) (Table 2).

**Table 2:** Respondents’ attitudes regarding the importance of an ICT research training course by current ICT using status

Items	All Respondents (*N* = 1065) Very important / Essential N (%)	Use ICT (*N* = 740) Very important / Essential N (%)	Not use ICT (*N* = 325) Very important / Essential N (%)	*p-*Value
a. An overview of ways that ICT can support/enhance the entire research process	720 (68)	518 (70)	202 (62)	0.231
b. Learning what researchers need to know about ICT to be efficient and effective researchers	756 (71)	544 (74)	212 (65)	0.006
c. Overview of types of ICT research methods/resources for researchers	692 (65)	486 (66)	206 (63)	0.470
d. Most useful ICT research methods/resources across the research process and where/how to access them	738 (69)	529 (71)	209 (64)	0.019
e. What is new/important in ICT research methods/resources that will help researchers make significant advances in their research	734 (69)	512 (69)	222 (68)	0.775
f. Developing an overall plan for learning about and using ICT research methods/resources in your research projects	708 (66)	497 (67)	211 (65)	0.476
g. Best ways to set up an experiment using ICT research methods/resources before generating data	722 (68)	511 (69)	211 (65)	0.184
h. Fundamentals of how databases are structured, attributes of data, ways data can be stored/retrieved and how pieces of data relate to each other	711 (67)	512 (69)	199 (61)	0.011
i. Principles, best practices and challenges of accessing/using existing data in clinical and population health research	683 (64)	480 (65)	203 (62)	0.451
j. What kinds of databases exist, how to access them and possible new uses of these data in research	705 (66)	503 (68)	202 (62)	0.065
k. Creating new databases, determining what data to collect, how to represent the data, how pieces of data need to relate to each other (i.e. data modeling)	699 (66)	486 (66)	213 (66)	0.965
l. Principles and challenges when integrating or using multiple datasets	629 (59)	430 (58)	199 (61)	0.340
m. Using data from electronic health records in research	698 (66)	500 (68)	198 (61)	0.036
n. Informatics principles and electronic methods to collect/capture data in clinical settings, including use of specific devices like Smart phones	689 (65)	488 (66)	201 (62)	0.197
o. Assuring data quality and fidelity when using electronic methods to acquire/ capture data	766 (72)	544 (73)	222 (68)	0.082
P. The concept and importance of data standards	704 (66)	493 (67)	211 (65)	0.590
q. Issues of security, ethical, legal, regulatory and confidentiality considerations in accessing/ using data	740 (69)	520 (70)	220 (68)	0.400
r. Best practices for managing data, including storage, security and retrieval	729 (68)	512 (69)	217 (67)	0.434
s. Using informatics methods to manipulate and analyze data, including understanding what you can and cannot do with data	745 (70)	526 (71)	219 (67)	0.226
t. Choosing the best ways to analyze specific data and address specific research questions	754 (71)	530 (72)	224 (69)	0.372
u. Tools and approaches for interpreting results, such as software to help visualize data	732 (69)	520 (70)	212 (65)	0.102
v. Conducting systematic data searches (e.g., literature reviews, vetted sources of information)	739 (69)	525 (71)	214 (66)	0.096
w. Using informatics search methods to help develop research protocols	688 (65)	495 (67)	193 (59)	0.018
x. Using information technology-based methods to recruit human subjects	626 (59)	440 (59)	186 (57)	0.496
y. Using information technology-based methods to develop and implement clinical decision support tools	660 (62)	459 (62)	201 (62)	0.955
z. Seeking help - kinds of informatics help you can get, knowing when to ask for help, kinds of people to approach for help with various types of issues/questions	705 (66)	497 (67)	208 (64)	0.315
aa. Using ICT to deliver clinical and population health interventions	700 (66)	493 (67)	207 (64)	0.354
bb. Collaborating effectively with informatics/ IT consultants and experts (i.e. knowing how to ask for what you want)	733 (69)	513 (69)	220 (67)	0.596
cc. Performing personal “computer hygiene” (backing up files, organizing files, archiving email)	762 (72)	546 (74)	216 (66)	0.015

### Practices regarding ICT

Of the 1065 respondents, 45% thought that “Give presentations in the universities on key ICT-related topics and the use of ICT by faculty researchers, educators and clinical service directors” might be the best way to reach academic healthcare faculty with the marketing or promotional messages of the ICT education program. Only 20% thought “Promote the value of ICT education of students, faculty and other staff at the university as an important component of research capacity-building” might be the best way, but ICT users compared to non-users were more likely to agree to this (*p* <  0.05) (Table 3).

**Table 3:** Respondents’ practices regarding the importance of an ICT research training course by ICT using status

Items	All Respondents (*N* = 1065) Very important / Essential N (%)	Use ICT (*N* = 740) Very important / Essential N (%)	Not use ICT (*N* = 325) Very important / Essential N (%)	*p-*Value
What might be the best ways to reach academic healthcare faculty at your university or other universities in China with the marketing or promotional messages of the ICT education program?				
a. To persuade the first groups of faculty who take the ICT course and apply it to their academic work to share their personal experiences and achievements with other faculty at their universities.	242 (23)	164 (22)	78 (24)	0.510
b. Give presentations at GXMU and other target universities on key ICT-related topics and the use of ICT by faculty researchers, educators and clinical service directors at their institutions and at other institutions in China and elsewhere.	474 (45)	326 (44)	148 (46)	0.654
c. Publicize the course(s) and their “results” through electronic based academic communications and other means to faculty, students, and staff of the University and its affiliated hospital(s) and other healthcare delivery entities.	131 (12)	86 (12)	45 (14)	0.309
d. Promote the value of ICT education of students, faculty and other staff at the university as an important component of research capacity-building.	218 (20)	164 (22)	54 (17)	0.039
Which of the below ICT and/or biomedical informatics tools do you use or plan to use? (*multiple choice*)				
a. Mobile phone	848 (80)	613 (83)	235 (72)	< 0.001
b. Non-mobile computer system	460 (43)	351 (47)	109 (34)	< 0.001
c. Radio	59 (6)	34 (5)	25 (8)	0.042
d. Internet	626 (59)	457 (62)	169 (52)	0.003
e. Cable TV	92 (9)	62 (8)	30 (9)	0.648
f. CD-ROM	63 (6)	44 (6)	19 (6)	0.949
g. WeChat	467 (44)	338 (46)	129 (40)	0.070
h. QQ	321 (30)	240 (32)	81 (25)	0.014
i. Skype	40 (4)	30 (4)	10 (3)	0.440
j. Others	10 (1)	5 (1)	5 (2)	0.179
What aspect of your professional work would you like to consider using more ICT and/or biomedical informatics methods? (*multiple choice*)				
a. Support for information seeking or decision-support by medical/healthcare providers	688 (65)	499 (67)	189 (58)	0.004
b. Support for health information or healthcare advice by patients/laymen	519 (49)	366 (49)	153 (47)	0.474
c. Help in identifying and/or implementing ICT and/or biomedical informatics method(s) to deliver a healthcare intervention	554 (52)	388 (52)	166 (51)	0.683
d. Help in using ICT and/or biomedical informatics to deliver healthcare in urban/suburban settings	379 (36)	278 (38)	101 (31)	0.042
e. Help in using ICT and/or biomedical informatics to deliver healthcare in remote or rural settings	315 (30)	240 (32)	75 (23)	0.002
f. Help in using ICT and/or biomedical informatics to monitor health and healthcare interventions in urban/suburban settings	286 (27)	210 (28)	76 (23)	0.090
g. Help in using ICT and/or biomedical informatics to monitor health and healthcare in rural or remote settings	273 (26)	202 (27)	71 (22)	0.061
h. Support on using ICT and/or biomedical informatics methods in my research	293 (28)	229 (31)	64 (20)	< 0.001
i. Support for gathering data for research from research subjects	289 (27)	220 (30)	69 (21)	0.004
j. Facilitating collaboration with research/clinical team members and other individuals	251 (24)	194 (26)	57 (18)	0.002
Was the ICT/biomedical informatics training that you have received helpful for your research or education? ^a^				
Very helpful	51 (71)	49 (88)	2 (13)	< 0.001
Somewhat helpful/ Not at all helpful	21 (29)	7 (12)	14 (87)	
In general, how helpful was the online course or courses that you have taken? ^b^				
Very helpful	136 (51)	119 (82)	17 (14)	< 0.001
Somewhat helpful/ Not at all helpful	133 (49)	26 (18)	107 (86)	

^a^*N* = 72, ^b^*N* = 269

Of the 740 users of ICT for research and/or training, more than 80% used or planned to use mobile phone, 47% used or planned to use non-mobile computer system, 62% used or planned to use Internet, and 32% used or planned to use QQ (a popular chat tool in China) in their current research activities, which were significantly higher than whom did not use ICT (all *p* <  0.05) (Table 3).

Compared to those who did not use ICT for research and/or training, ICT users were more likely to consider using more ICT and/or biomedical informatics methods in “Support for information seeking or decision-support by medical/healthcare providers”, “Help in using ICT and/or biomedical informatics to deliver healthcare in urban/suburban settings”, “Help in using ICT and/or biomedical informatics to deliver healthcare in remote or rural settings”, “Support on using ICT and/or biomedical informatics methods in my research”, “Support for gathering data for research from research subjects”, and “Facilitating collaboration with research/clinical team members and other individuals” (all *p* <  0.05) (Table 3).

More than 80% of the ICT users expressed the opinions that ICT/biomedical informatics training and the online courses were very helpful, which were significantly higher than whom did not use ICT (both *p* <  0.001) (Table 3).

## Discussion

This study examined the attitudes and practices of health sciences postgraduates regarding curriculum for ICT use in global health research and training at six medical universities in southern China. The results of this study revealed that currently ICT use for research and/or training is low amongst the health sciences postgraduates: 90% reporting they did not have training in any aspect of ICT or biomedical informatics for use in research related work, three quarters reporting they have not taken any online course; only 69% have used ICT in their current research activities. In contrast, in Uganda, 94% of the health sciences students reported using ICT for research/training purposes [14]. These differences in ICT use patterns may be due to the variations in defining ICT use between our study and the study in Uganda [14]. As the term ICT is vaguely used across studies, future studies should clearly define the scopes of activities that are covered to describe ICT. Consistent with the findings in another study in Uganda, participants in our study revealed that ICT use was a potentially important tool for their education and research [14]. This underscore the increased awareness of ICT among postgraduates which is linked to the growing use of technology in the healthcare delivery, including patient education, training of healthcare providers and monitoring of healthcare services [15–17].

Several earlier studies described the importance of ICT knowledge and skills among the medical students. These studies reported that to enhance their ability to acquire, appraise, and solve clinical and other problems, medical students strongly felt ICT skills training should be a part of medical curriculum and teaching of ICT skills should be integrated into medical studies [18–22]. Supporting these findings, in our study, about 67% of the health sciences postgraduates expressed the opinion that knowledge of databases, ways of data acquisition, and using data from electronic health records in research were very important or essential for their educational needs. In a capacity building project in Kuwait, after the ICT training curriculum, most of the students’ ICT skills were improved and research activities increased [23], underscoring the usefulness of training curricula. As the training of data collection methods and data analysis software, such as SAS, SPSS, and GIS (geographic information systems), has been identified as an important component for ICT training by health sciences postgraduates and faculty [1, 11, 24], future ICT training curricula should consider this in the planning and implementation of such program.

About half of our participants thought “Giving presentations on key ICT-related topics and the use of ICT” might be the best way to reach academic healthcare faculty with the marketing or promotional messages of the ICT education program because health sciences postgraduates believed that an ICT curriculum with specific and pertinent topics related to their major would be more welcomed [11]. Presentations on the use of ICT by faculty researchers, educators and clinical service directors would supply references and success stories, which may improve students’ learning interest and confidence.

We also found that 80% of the respondents used or planned to use mobile phones, 59% used or planned to use Internet, more than 40% used or planned to use computer and WeChat (the most popular social network tool in China), and 30% used or planned to use QQ as ICT and/or biomedical informatics tools in their current research activities. The utilization rate of mobile phone in Chinese college students was very high, and almost all of the mobile phones used by young people were smartphones with the function of accessing Internet, which meant that mobile phones and good network coverage might make this a viable strategy for leveraging mobile ICT for health education and research [14]. A study conducted in Uganda showed that most of the health sciences students in Makerere University used their mobile phones for education and research, and 94% of the students centered on using the Internet for research/learning purposes [14]. In southern China, more than 62% of health sciences graduate students accessed Internet frequently to check their emails [11]. Computers were also a tool with very high utilization rate used by health sciences postgraduates in China. Their main computer activities were electronic document processing, Internet searching, educational or research materials downloading, data reduction and analyses, and online chat [14, 25]. In China, WeChat and QQ were the most popular social tools could be operated on a computer or mobile phone when linked to Internet. Through these two ICT tools with Internet, researchers could conduct different aspects of global health research, deliver healthcare services, and monitor health systems.

Our study findings showed that, compared to whom did not use ICT, the ICT users were more likely to consider using more ICT and/or biomedical informatics methods in decision-support or support for information seeking, healthcare delivery, academic research, data gathering, and facilitating research collaboration. ICT could directly link health and medical professionals in developing countries with experts in the developed countries, had the potential to increase access to training in global health research through online courses, so that health and medical professionals might solve the local health problems through information exchange and telemedicine consultation [1, 26].

### Limitations

This study had several limitations. First, the sample may not be representative of the whole health sciences postgraduate student population in China. Secondly, all the data were collected by an online questionnaire survey platform, without having any opportunity for the participant to clarify any issue. Thirdly, participants responded to the online survey through an open call in the participating universities, so we are unable to calculate the response rate.

## Conclusions

This study examined health sciences postgraduates’ attitudes and practices regarding curriculum for ICT use in global health research and training in China. It identified potential curriculum contents that should be included in future ICT research training curriculum. This also documented which ICT and/or biomedical informatics tools that health sciences postgraduates used or planned to use in their research activities. Additionally, the study examined in which aspects of professional work health sciences postgraduates would like to consider using more ICT and/or biomedical informatics methods. To better understand health sciences postgraduates’ attitudes and practices regarding ICT use, a further nation-wide survey is needed across medical universities throughout China. Nevertheless, the findings of the current study should provide educators and researchers some initial insights that could be incorporated within the framework of any future ICT training programs.

## Additional file

Additional file 1: Questionnaire used in the online survey. (DOC 78 kb)

## Data Availability

The datasets used and/or analysed during the current study available from the corresponding author on reasonable request. All data generated or analysed during this study are included in this published article.

## Abbreviations

FDU: Fudan University
GIS: Geographic Information Systems
GLMU: Guilin Medical University
GXMU: Guangxi Medical University
GXUCM: Guangxi University of Chinese Medicine
GZMU: Guangzhou Medical University
ICT: Information and Communication Technology
KMMU: Kunming Medical University
