# Supplementary material for: Attitudes of Chinese health sciences postgraduate students’ to the use of information and communication technology in global health research
Source: BMC Med Educ. 2019 Oct 9;19:367. doi: 10.1186/s12909-019-1785-6 (PMC6784339; doi:10.1186/s12909-019-1785-6)
Supplement: Supplementary file 1 — Questionnaire used in the online survey. (DOC 78 kb) [file 12909_2019_1785_MOESM1_ESM.doc]

**Needs Assessment Survey**

**[Graduate student]**

**Questionnaire for Basic Science, Clinical, and Population Health Researchers**

Thank you for answering the questions in this questionnaire about a possible plan to develop a brief and efficient educational program in the use of information and communication technologies (ICT) by basic, clinical and population health researchers at health-related universities in China. The curricula will be developed by six major Universities in South China including Shanghai Fudan University, Kunming University, Guangxi Medical University, Guangxi Chinese Medicine University, Guilin Medical University and Guangzhou Medical University in collaboration with Boston University’s Medical Information Systems Unit (MISU). The specific goal of the program is to create a model set of easy-to-use curricula to train busy researchers at Guangxi Medical University (GMU) and other Chinese universities about ICT research methods that they can employ successfully in their research.

For your reference, Information and Communications Technology (ICT) can be defined as a collection of technologies and applications that are used to process, store and disseminate information to a wide variety of users or clients. ICT can play a critical role in improving population health and delivery of health care for individuals and communities. The use of ICT in the health sciences filed can be categorized into 4 main areas: Health Education, Hospital Management System, Health Research, and Health Data Management.

We appreciate your thoughts and comments in this needs assessment survey as you and other researchers working in health sciences universities in China will personally benefit from your comments and suggestions!

**SECTION A:** CONTENT RATINGS AND SUGGESTIONS

- *In this section, we request your thoughts about the content of an ICT related research training curriculum for faculty researchers at several Medical Universities in China*
- *Please give response based on your knowledge, experience and/or expectations for ICT education/training for faculty researchers*
- *Please understand that some content from one curriculum item may overlap with some of the content in another item.*

**A1. Rate each of the following curriculum items for how important you consider them to be in an ICT research training course for you.**

|  | Not at all important | A little important | Somewhat important | Very important | Essential |
| --- | --- | --- | --- | --- | --- |
| a. An overview of ways that ICT can support/enhance the entire research process |  |  |  |  |  |
| b. Learning what researchers need to know about ICT to be efficient & effective researchers |  |  |  |  |  |
| c. Overview of types of ICT research methods/resources for researchers |  |  |  |  |  |
| d. Most useful ICT research methods/resources across the research process & where/how to access them |  |  |  |  |  |
| e. What is new/important in ICT research methods/resources that will help researchers make significant advances in their research |  |  |  |  |  |
| f. Developing an overall plan for learning about & using ICT research methods/resources in your research projects |  |  |  |  |  |
| g. Best ways to set up an experiment using ICT research methods/resources before generating data |  |  |  |  |  |
| h. Fundamentals of how databases are structured, attributes of data, ways data can be stored/retrieved & how pieces of data relate to each other |  |  |  |  |  |
| i. Principles, best practices & challenges of accessing/using existing data in clinical & popopulation health research |  |  |  |  |  |
| j. What kinds of databases exist, how to access them & possible new uses of these data in research |  |  |  |  |  |
| k. Creating new databases, determining what data to collect, how to represent the data, how pieces of data need to relate to each other (i.e. data modeling) |  |  |  |  |  |
| l. Principles & challenges when integrating or using multiple datasets |  |  |  |  |  |
| m. Using data from electronic health records in research |  |  |  |  |  |
| n. Informatics principles & electronic methods to collect/capture data in clinical settings, including use of specific devices like Smartphones |  |  |  |  |  |
| o. Assuring data quality & fidelity when using electronic methods to acquire/ capture data |  |  |  |  |  |
| P.The concept & importance of data standards |  |  |  |  |  |
| q. Issues of security, ethical, legal, regulatory & confidentiality considerations in accessing/ using data |  |  |  |  |  |
| r. Best practices for managing data, including storage, security & retrieval |  |  |  |  |  |
| s. Using informatics methods to manipulate & analyze data, including understanding what you can & cannot do with data |  |  |  |  |  |
| t. Choosing the best ways to analyze specific data & address specific research questions |  |  |  |  |  |
| u. Tools & approaches for interpreting results, such as software to help visualize data |  |  |  |  |  |
| v. Conducting systematic data searches (e.g., literature reviews, vetted sources of information) |  |  |  |  |  |
| w. Using informatics search methods to help develop research protocols |  |  |  |  |  |
| x. Using information technology-based methods to recruit human subjects |  |  |  |  |  |
| y.Using information technology-based methods to develop & implement clinical decision support tools |  |  |  |  |  |
| z. Seeking help - kinds of informatics help you can get, knowing when to ask for help, kinds of people to approach for help with various types of issues/questions |  |  |  |  |  |
| aa. Using ICT to deliver clinical & population health interventions |  |  |  |  |  |
| bb. Collaborating effectively with informatics/ IT consultants and experts (i.e. knowing how to ask for what you want) |  |  |  |  |  |
| cc. Performing personal “computer hygiene” (backing up files, organizing files, archiving email) |  |  |  |  |  |

A2**. Are there any essential content areas missing? If so, please explain.**

_______________________________________________________________

_______________________________________________________________

_______________________________________________________________

**A3. If you had time to ATTEND class to learn about ICT, please list up to five topics that you would like to learn about, starting with the most important one.**

(a) Most Important Topic to LEARN: ___________________________________________

(b) 2^nd^ Most Important Topic to LEARN: _________________________________________

(c) 3^rd^ Most Important Topic to LEARN: _________________________________________

(d) 4^th^ Most Important Topic to LEARN: _________________________________________

(e) 5^th^ Most Important Topic to LEARN: _________________________________________

**A4. If you were going to attend a course on ICT for clinical or population health researchers, what types of educational format(s) would you prefer?** *Put a percentage of TOTAL class AND homework time that used EACH format (from 0-100%, with the total for ALL formats totaling 100%).*

(a) Classroom-based lecture:_________________________________ : ---%

(b) Classroom-based small seminar:___________________________ : ---%

(c) Online presentations with access to the material via flexible tools : ---%

(d) Classroom-based workshops:____________________________ : ---%

(e) Classroom-based small group discussion:____________________: ---%

(f) Problem based learning:_____________ ____________________ : ---%

(g) Interactive teaching:____________________________________ : ---%

(h) Interactive learning: ____________________________________ : ---%

**Total:** _____________________________________________ : **100%**

**A5. How much time do you think that a typical graduate student or researcher would be willing to spend to learn ICT to help him/her be a more effective and efficient researcher?** *Give the minimum and maximum number of hours.*

Minimum number of hours: ________ hours

Maximum number of hours: ________ hours

**A6. Are there any challenges or issues you can think of in terms of learning about ICT that would need to be addressed in such a curriculum?**

(a) Yes (specify), _______________________________

(b) No

**A7. What marketing messages or means of marketing the ICT program/curriculum might appeal to academic healthcare faculty researchers, educators and clinicians?** *Check all that apply.*

1. Examples of academic health faculty researchers, educational program directors/education faculty & directors of healthcare service programs who have successfully used ICT in their work.
2. Examples of academic healthcare faculty (see in item (a) above) who learned how to use ICT in their work from a formal ICT education program.
3. Examples of time efficient & convenient access to ICT educational programs for busy academic health faculty.
4. Examples of programs that teach academic healthcare faculty skills, etc. in which the faculty who attend the program obtain academic credit and/or work performance credit for taking the academic skill-development course and/or of demonstrating that they used the training in their academic work.
5. Others: (please specify): _____________________________________

**A8. What might be the best ways to reach academic healthcare faculty at your university or other universities in China with these marketing or promotional messages of the ICT education program?** *Check all that apply.*

1. To persuade the first groups of faculty who take the ICT course and apply it to their academic work to share their personal experiences and achievements with other faculty at their universities.
2. Give presentations at GXMU & other target universities on key ICT-related topics and the use of ICT by faculty researchers, educators and clinical service directors at their institutions and at other institutions in China and elsewhere..
3. Publicize the course(s) and their “results” through electronic-based academic communications and other means to faculty, students, and staff of the University and its affiliated hospital(s) & other healthcare delivery entities.
4. Promote the value of ICT education of students, faculty and other staff at the university as an important component of research capacity-building
5. Develop and implement ICT research projects at the University as a showcase of the use & value of ICT in research & other endeavors at the healthcare university
6. Others: (Please specify): _____________________________________

**SECTION B**: OTHER THOUGHTS AND COMMENTS

**B1. One possible design for these training curriculum for researchers is to offer an initial overview of ICT use and informatics for researchers (what it is, what it can do for the researcher, some basic concepts that all researchers should know), followed by a series of “electives” on specific topics, designed to be taught in a very short amount of time. In your opinion, how effective could this format be for covering the material you believe should be taught?**

| Very Ineffective |  |  |  | Very effective |
| --- | --- | --- | --- | --- |
| 1 | 2 | 3 | 4 | 5 |

**B2. What other thoughts/suggestions do you have regarding the curriculum for short courses in ICT or medical informatics for clinical and population researchers, and/or behavioral/social scientists, including information about existing courses/programs?**

_______________________________________________________________

__________________________________________________________________

___________________________________________________________________

__________________________________________________________________

**SECTION C**: BACKGROUND INFORMATION

**C1. What is your institutional affiliation?**

(a) Guangxi Medical University

(b) Guangxi University of Chinese Medicine

(c) Guilin Medical University

(d) Guangzhou Medical University

(e) Kunming Medical University

(f) Shanghai Fudan University

(g) Other (please specify): ______________________

**C2. With which school or academic center are you affiliated with?**

(a) School of Medicine

(b) School of Public Health

(c) School of Pharmacy

(d) School of Nursing

(e) School of Medical Informatics

(f) Others (please specify):_________________________

**C3. What is your current position?**

(a) Masters student

(b) Doctoral student

(c) Post-graduate trainee

(d) Others (please specify):_______________

**C4. What kind of research do you primarily perform or hope to perform?** *Check all that apply.*

(a) None (SKIP TO **Question#C6**)

(b) Biomedicine

(c) Clinical Medicine

(d) Health Communication

(e) Biomedical, Medical or Public Health Informatics (or another healthcare informatics)

(f) Population health and/or public health research

(g) Epidemiology/clinical epidemiology

(h) Health services research

(i). Behavioral/social science research

(k).Other (specify): __________________________________

**C5. How would you rate your level of knowledge of the use of biomedical informatics in the primary type of research you selected in question #C4 above?**

Very low Very high

1 2 3 4 5 6 7 8 9 10

**C6. What is/are your role(s) in relationship to the use of ICT in biomedical/health research?** *Check all that apply.*

(a) Teach ICT &/or biomedical informatics to biomedical researchers or biomedical researchers in training

(b) Develop courses/curricula in ICT &/or biomedical informatics

(c) Consult with/help researchers use ICT &/or biomedical informatics

(d) Develop new tools/methods in ICT &/or biomedical informatics

(e) Do research on ICT &/or biomedical informatics

(f) Use ICT &/or biomedical informatics in my research

(g) Plan to use ICT &/or biomedical informatics in my research

(h) Other: please specify___________________________________

**C7. How many years have you been involved in health-related research?**

__________ years.

**C8. Do you use any ICT &/or biomedical informatics tools in your current research activities?**

(a) Yes

(b) No

(c) I plan to use ICT &/or biomedical informatics in my future research

**C9. Which of the below ICT &/or biomedical informatics tools do you use or plan to use? [Answer all those apply]**

(a) Mobile phone

(b) Non-mobile computer system

(c) Radio

(d) Internet

(e) Cable TV

(f) CD-ROM

(g) We chat

(h) QQ

(i) Skype

h) Others (specify):___________

**C10. What aspect of your professional work would you like to consider using more ICT &/or biomedical informatics methods?** Multiple choice*.*

(a) Support for information seeking or decision-support by medical/healthcare providers

(b) Support for health information or healthcare advice by patients/laymen

(c) Help in identifying &/or implementing ICT &/or biomedical informatics method(s) to deliver a healthcare intervention

(d) Help in using ICT &/or biomedical informatics to deliver healthcare in urban/suburban settings

(e) Help in using ICT &/or biomedical informatics to deliver healthcare in remote or rural settings

(f) Help in using ICT &/or biomedical informatics to monitor health & healthcare interventions in urban/suburban settings

(g) Help in using ICT &/or biomedical informatics to monitor health & healthcare in rural or remote settings

(h) Support on using ICT &/or biomedical informatics methods in my research

(i) Support for gathering data for research from research subjects

(j) Facilitating collaboration with research/clinical team members & other individuals

**C11. Have any of you had training, either formal or informal, in any aspect of ICT or biomedical informatics？**

1. Yes; What was the training about (please specify): _________________________________________________________

(b) No (go to Question **C14)**

**C12. Was the ICT/biomedical informatics training that you have received helpful for your research or education?**

(a) Very helpful

(b) Somewhat helpful

(c) Not at all helpful (go to Question **C14**)

**C13. Which aspect of the training that you have received was helpful?**

*Check all that apply.*

(a) Developing courseware

(b) Preparing power point presentation

(c) Using MOOCs (Massive Open Online Courses)

(d) Use of computer programming language (i.e. JAVA, VB, Access)

(e) Understanding of research analytical methods (i.e., biomedical statistics)

(f) Document information retrieval

(d) Others (Please specify): ________________________________

**C14. Have you ever taken an online course?**

(a) Yes; how many (please specify):______ on-line courses

(b) No (go to Question **C17**)

**C15. In general. how helpful was the online course or courses that you have taken?**

(a) Very helpful

(b) Somewhat helpful

(c) Not at all helpful (go to Question **C17**)

**C16. Why do you think that the online course you took was helpful?**

*Check all that apply.*

(a) It was convenient

(b) Helped me understand a topic of interest to me

(c) Help me apply useful methods in my work

(d) Others, (Please specify): ________________________________

**C17. What is your gender?**

(a) Female (b) Male

**C18. What is your age?** _______ (Years)

**C19. Do you have other comments?**

____________________________________________________________

____________________________________________________________

**20. Do you want to take part in future research about the use of ICT in population health or global health research? If so, please write down your e-mail address or qq address?**

____________________________________________________________

**THANK YOU for participating in this needs assessment.**
